# Examination of the Safety of Pediatric Vaccine Schedules in a Non-Human Primate Model: Assessments of Neurodevelopment, Learning, and Social Behavior

**DOI:** 10.1289/ehp.1408257

**Published:** 2015-02-18

**Authors:** Britni Curtis, Noelle Liberato, Megan Rulien, Kelly Morrisroe, Caroline Kenney, Vernon Yutuc, Clayton Ferrier, C. Nathan Marti, Dorothy Mandell, Thomas M. Burbacher, Gene P. Sackett, Laura Hewitson

**Affiliations:** 1 Infant Primate Research Laboratory, Washington National Primate Research Center, and; 2 Center on Human Development and Disability, Seattle, Washington, USA; 3 Abacist Analytics, LLC, Austin, Texas, USA; 4 Independent Consultant, Austin, Texas, USA; 5 Department of Environmental and Occupational Health Sciences, and; 6 Department of Psychology, University of Washington, Seattle, Washington, USA; 7 The Johnson Center for Child Health and Development, Austin, Texas, USA; 8 Department of Psychiatry, University of Texas Southwestern, Dallas, Texas, USA

## Abstract

**Background:**

In the 1990s, the mercury-based preservative thimerosal was used in most pediatric vaccines. Although there are currently only two thimerosal-containing vaccines (TCVs) recommended for pediatric use, parental perceptions that vaccines pose safety concerns are affecting vaccination rates, particularly in light of the much expanded and more complex schedule in place today.

**Objectives:**

The objective of this study was to examine the safety of pediatric vaccine schedules in a non-human primate model.

**Methods:**

We administered vaccines to six groups of infant male rhesus macaques (*n* = 12–16/group) using a standardized thimerosal dose where appropriate. Study groups included the recommended 1990s Pediatric vaccine schedule, an accelerated 1990s Primate schedule with or without the measles–mumps–rubella (MMR) vaccine, the MMR vaccine only, and the expanded 2008 schedule. We administered saline injections to age-matched control animals (*n* = 16). Infant development was assessed from birth to 12 months of age by examining the acquisition of neonatal reflexes, the development of object concept permanence (OCP), computerized tests of discrimination learning, and infant social behavior. Data were analyzed using analysis of variance, multilevel modeling, and survival analyses, where appropriate.

**Results:**

We observed no group differences in the acquisition of OCP. During discrimination learning, animals receiving TCVs had improved performance on reversal testing, although some of these same animals showed poorer performance in subsequent learning-set testing. Analysis of social and nonsocial behaviors identified few instances of negative behaviors across the entire infancy period. Although some group differences in specific behaviors were reported at 2 months of age, by 12 months all infants, irrespective of vaccination status, had developed the typical repertoire of macaque behaviors.

**Conclusions:**

This comprehensive 5-year case–control study, which closely examined the effects of pediatric vaccines on early primate development, provided no consistent evidence of neurodevelopmental deficits or aberrant behavior in vaccinated animals.

**Citation:**

Curtis B, Liberato N, Rulien M, Morrisroe K, Kenney C, Yutuc V, Ferrier C, Marti CN, Mandell D, Burbacher TM, Sackett GP, Hewitson L. 2015. Examination of the safety of pediatric vaccine schedules in a non-human primate model: assessments of neurodevelopment, learning, and social behavior. Environ Health Perspect 123:579–589; http://dx.doi.org/10.1289/ehp.1408257

## Background

During the 1990s, thimerosal, an ethylmercury (EtHg)–based preservative, was included in several vaccines given to U.S. infants (Clements et al. 2000). Many infants received up to 187.5 μg EtHg by 6 months of age by following the recommended pediatric vaccination schedule (Pichichero et al. 2008). This cumulative exposure exceeded the U.S. Environmental Protection Agency’s safe intake level, estimated in 1997 to be ≤ 0.1 μg of mercury/kg body weight (BW)/day (U.S. Environmental Protection Agency 1997). However, these safety recommendations are based on data from exposure to oral methylmercury (MeHg), not intramuscular (IM) EtHg. Some parent and advocacy groups raised concerns over a possible link between the use of EtHg in vaccines and the increasing rates of developmental disorders, which has in turn negatively impacted immunization rates (Biroscak et al. 2003). In 1999, the Centers for Disease Control and Prevention (CDC) and the American Academy of Pediatrics recommended that thimerosal be removed from pediatric vaccines (CDC 1999).

Since that time, the Advisory Committee on Immunization Practices has markedly expanded pediatric vaccination recommendations (Fiore et al. 2008). By 2008, multiple doses of rotavirus, hepatitis A, pneumococcal, varicella, and meningococcal vaccines, as well as a yearly influenza vaccine for all children 6 months to 18 years of age, had been added to the vaccine schedule. Despite the recommended removal of thimerosal from pediatric vaccines in the United States, multidose influenza and meningococcal vaccines still include thimerosal as a preservative (Food and Drug Administration 2012) and are administered to many infants and/or pregnant women (Dórea et al. 2013). Additional thimerosal-containing vaccines (TCVs), such as that for hepatitis B, are also administered to millions of children globally (Dórea et al. 2013). As the U.S. vaccine schedule has expanded, parental perceptions that vaccines pose safety concerns have grown (Gust et al. 2009; Kempe et al. 2011), especially since there have been no preclinical studies examining the safety of new pediatric vaccine schedules in their entirety before universal recommendation.

Much of the research examining the safety of pediatric vaccines is based on rodent data. Specifically, these studies have investigated potential neurobehavioral effects of prenatal and/or postnatal thimerosal exposure (Berman et al. 2008; Hornig et al. 2004; Laurente et al. 2007; Olczak et al. 2011; Sulkowski et al. 2012). At thimerosal doses equivalent to those previously present in pediatric vaccines, few, if any, neurobehavioral effects were identified (Berman et al. 2008). When an adverse effect was reported, it was typically when very high doses of thimerosal (as much as 250 times that found in vaccines) were used (Li et al. 2014; Olczak et al. 2011) and/or the route of exposure differed (Li et al. 2014; Sulkowski et al. 2012). Several studies have already established that oral treatment and IM injections with thimerosal in mice result in different toxicokinetics (Harry et al. 2004; Rodrigues et al. 2010), indicating that the route of administration is crucial in these studies. Furthermore, small improvements to experimental methodology, such as a reduction in injection volume (thereby avoiding possible hindlimb damage), resulted in a previously reported adverse neurobehavioral effect (Hornig et al. 2004) no longer being significant (Berman et al. 2008). Clearly, one must take into account the dose of thimerosal used, the route of administration, and the injection volume when reviewing the literature to avoid misinterpretation of the findings. Ultimately, although the rodent literature has helped inform us about experimental design for thimerosal studies, the small size of mouse pups represents significant challenges particularly when administering IM thimerosal (Harry et al. 2004).

With these limitations in mind, we developed a non-human primate model to examine the effects of different vaccine schedules on neurobehavioral development. Non-human primates (hereafter referred to as primates) share a great deal of evolutionary history with humans, and as such, are particularly relevant for neurobehavioral and neurocognitive evaluations. Questions addressing more complex cognitive processes and intricate social interactions may therefore be better suited for non-human primate studies (Nelson and Winslow 2009; Patten et al. 2014). Furthermore, primates are especially useful for studies of developmental exposures because they, like humans, have relatively prolonged periods of gestation, infancy, and adolescence (Rice 1987). This long period of vulnerability allows investigation of critical variables during sensitive periods of exposure. Moreover, the nervous system of primates is quite comparable to that of humans (Nelson and Winslow 2009) and often responds similarly to toxic insult (Burbacher and Grant 2000; Golub 1990; Rice 1987; Schneider et al. 2011). Because infant development in primates shares many parallels with that of humans, a wide range of neurobehavioral tests, adapted from assessments used with human infants, are routinely implemented for monitoring developmental trajectories in infant primates following exposure to environmental neurotoxicants (Burbacher and Grant 2000; Gunderson et al. 1988; Rice 1999; Rice and Hayward 1997).

In summary, primates provide a relevant animal model for exploring potential neurobehavioral consequences of environmental neurotoxicant exposures, such as thimerosal. In a controlled, blinded primate study, we examined the safety of pediatric vaccines, including TCVs, on a number of neurobehavioral tests: acquisition of neonatal reflexes, development of object permanence, formation of discrimination learning strategies, and assessments of social behavior.

## Materials and Methods

*Animal assurances*. Animal procedures followed the guidelines of the Animal Welfare Act and the *Guide for Care and Use of Laboratory Animals* (National Research Council 2011). The Washington National Primate Research Center (WaNPRC) and the University of Washington are fully accredited by the Association for Assessment and Accreditation of Laboratory Animal Care. The experimental design and research protocols were approved by the University of Washington Institutional Animal Care and Use Committee, and all animals were treated humanely and with regard for alleviation of suffering.

*Animal husbandry*. Rhesus macaque (*Macaca mulatta*) pregnancies were produced by natural mating at the California National Primate Research Center (CNPRC). We selected pregnant dams based on their overall health and confirmation of a male fetus of suitable gestational age by ultrasound. Prior pregnancy records were also reviewed to avoid nulliparous dams or dams with a history of miscarriage. Pregnant dams were transported from the CNPRC to the WaNPRC Infant Primate Research Laboratory (IPRL) by a specialized animal trucking company and monitored 24 hr/day using infrared cameras until delivery.

*Study design*. A total of 79 male infant macaques were studied in six groups (Table 1): *a*) control (animals received saline injections in place of vaccines); *b*) MMR (animals received only the MMR vaccine); *c*) TCV (animals received all TCVs but no MMR vaccines); *d*) 1990s Pediatric (animals received TCV and MMR vaccines following the pediatric schedule recommended in the 1990s); *e*) 1990s Primate (animals received all vaccines recommended in the 1990s but with the timing accelerated approximately 4:1); and *f* ) 2008 (animals received the expanded pediatric vaccine schedule that was in place in 2008, which remains very similar to the current recommended vaccine schedule).

**Table 1:** Study groups, sample sizes (n), and schedules for vaccine administration.

Group	*n*	Birth	2 weeks	4 weeks	6 weeks	15 weeks	52 weeks
Control	16		Saline	Saline	Saline	Saline	Saline
		Saline	Saline	Saline	Saline	Saline	Saline
			Saline	Saline	Saline	Saline
MMR	15		Saline	Saline	Saline	MMR	MMR
		Saline	Saline	Saline	Saline	Saline	Saline
			Saline	Saline	Saline	Saline
TCV	12		Hep B	Hep B	Hep B	Saline	Saline
		Hep B	DTaP	DTaP	DTaP	DTaP	DTaP
			Hib	Hib	Hib	Hib
1990s Primate	12	Hep B	Hep B	Hep B	Hep B	MMR	MMR
			DTaP	DTaP	DTaP	DTaP	DTaP
			Hib	Hib	Hib	Hib
1990s Pediatric^*a*^	12	Hep B	Hep B	Hep B	Hep B	MMR	None
			DTaP	DTaP	DTaP	DTaP
			Hib	Hib	Hib	Hib
2008	12	See Supplemental Material, Table S3, for details					
Abbreviations: Hep B, hepatitis B vaccine; DTaP, diphtheria, tetanus, acellular pertussis vaccine; Hib, Haemophilus influenza B vaccine; MMR, measles, mumps, rubella vaccine; TCV, thimerosal-containing vaccines. ^***a***^For the 1990s Pediatric group, vaccines were administered at birth, 2 months, 4 months, 6 months and 15 months; the MMR and DTaP boosters were not administered at 52 months because animals were sacrificed at approximately 18 months.							

We preassigned infants to a study group prior to delivery to distribute them across multiple study groups within a single breeding season (see Supplemental Material, Table S1). Within each study group, infants were further assigned to a peer group such that their birth dates were within 30 days of each other. The only exception was in one of the four MMR peer groups, for which only three male infants within the appropriate age range were available. Gestational age and birth weight of all infants were within the normal range [mean ± SD gestational age, 166.8 ± 4.9 days; 95% confidence interval (CI): 153, 174 days; mean ± SD birth weight, 557.4 ± 72.7 g; 95% CI: 410, 780 g], with no statistically significant group differences (*p* > 0.05). Each infant received standard neonatal care and was raised during infancy in their individual home cage in the same rearing room as the other members of their peer group following standardized protocols (Sackett et al 2006a; Schneider and Suomi 1992).

*Vaccine source and dosing*. The source of vaccines and EtHg content for all vaccines used in this study are shown in Supplemental Material, Table S2. The recommended 1994–1999 U.S. pediatric immunization schedule included hepatitis B (Hep B); diphtheria, tetanus, acellular pertussis (DTaP); Haemophilus influenzae B (Hib); measles, mumps, rubella (MMR); and an oral polio vaccine. The Hep B, DTaP, and Hib vaccines available during that time contained thimerosal, an EtHg-based preservative. The MMR vaccine has always been thimerosal-free. To recreate the TCVs for this study, we purchased single-dose, thimerosal-free vaccines from the manufacturers listed in Supplemental Material, Table S2, and added thimerosal. To calculate the thimerosal content for each vaccine, we first determined the amount of EtHg (micrograms) administered to a male human infant in the 10th percentile for weight at the recommended times of vaccination (Table 2). Using the weights of male infant macaques on the 95th percentile (Ruppenthal 1985), we calculated the weight ratio for male human infants:male primate infants at each scheduled vaccination. This maximized possible infant exposure to thimerosal while still maintaining an appropriate clinical exposure. An average weight ratio of 6.3:1 for human:primate infants across the entire study period was used to calculate the final dosing of the TCVs. Standardization of thimerosal content for each vaccine across the study also allowed for valid comparison of outcomes and minimized errors in vaccine dosing.

**Table 2:** Primate equivalents of dosing and timing of the U.S. pediatric vaccine recommendations in the 1990s

	Birth	2	4	6	15	48
Humans [age (months)]
EtHg in vaccines (μg)
Hepatitis B × 3 doses	12.5	12.5	12.5	—	—	—
DTaP × 5 doses	—	25	25	25	25	25
Hib × 4 doses	—	25	25	25	25	—
MMR × 2 doses	—	—	—	—	0	0
Total EtHg for infant boys (μg)	12.5	62.5	62.5	50	50	25
10th percentile weights for infant boys (kg)^*a*^	2.8	4.4	5.8	6.8	9	14
EtHg for infant boys (μg/kg BW)	4.46	14.20	10.78	7.35	5.56	1.79
Primate [age (weeks)]
95th percentile weights for infant primates (kg)^*b*^	0.62	0.73	0.84	0.94	1.20	2.47
Weight ratio (infant boys:primates)	4.52	6.03	6.90	7.23	7.50	5.67
EtHg in vaccines (μg)^*c*^
Hepatitis B × 3 doses	1.98	1.98	1.98	—	—	—
DTaP × 5 doses	—	3.96	3.96	3.96	3.96	3.96
Hib × 4 doses	—	3.96	3.96	3.96	3.96	—
MMR × 2 doses	—	—	—	—	0	0
Total EtHg for primate vaccines (μg)	1.98	9.9	9.9	7.92	7.92	3.96
EtHg/kg for primates (μg/kg BW)	3.20	13.59	11.81	8.44	6.61	1.61
^***a***^Based on 10th percentile weights for infant boys from the weight-for-age percentiles from the National Center for Health Statistics (2001). ^***b***^Based on 95th percentile weights for infant male macaques (Ruppenthal 1985). ^***c***^EtHg content of primate vaccines was determined by first averaging the weight ratios for human infant boys:male infant primates across the six time points of vaccine administration; this yielded an average weight ratio of 6.3:1. The EtHg content in each pediatric vaccine was then divided by 6.3 to determine the dosing of EtHg for each primate vaccine. This provided a similar dosing of μg EtHg/kg BW for infant boys and primates.						

The preparation of TCVs and all quality assurance/quality control were performed at the University of Kentucky Environmental Research and Training Laboratory. Briefly, purchased vaccines were pooled prior to thimerosal addition. Stock thimerosal (T5125; Sigma-Aldrich) solutions were prepared such that a 50-μL dose added to the pooled vaccines would yield the desired EtHg concentrations. Triplicate stock thimerosal solutions and spiked vaccine solutions were digested in 5% nitric acid at 100^o^C for 2 hr and analyzed for EtHg concentration using a Varian Vista Pro CCD ICP-OES (simultaneous inductively coupled plasma optical emission spectrometer) to verify that target concentrations were achieved. Matrix effects were evaluated and corrected for using an yttrium internal standard. Furthermore, second-source curve verifiers and spike recoveries were > 95%. Laboratory control samples consisting of three different dilutions of the stock solutions bracketing the expected concentrations of the dosed vaccines were also prepared and analyzed alongside the dosed vaccines on a Nippon MA-2000 mercury analyzer (Nippon Instruments Corporation). Recoveries on the laboratory control samples were again > 95%. The TCVs contained either 1.98 μg EtHg per 0.5 mL dose (Hep B) or 3.96 μg EtHg per 0.5 mL dose (DTaP and Hib). We periodically verified the concentration of EtHg in vaccine aliquots throughout the study using an independent testing laboratory (Quicksilver Scientific).

For the 2008 schedule, additional vaccines were purchased from the manufacturers listed in Supplemental Material, Table S2. These included rotavirus, pneumococcal, inactivated polio virus, varicella, hepatitis A, meningococcal, and influenza vaccines, which were administered according to the schedule listed in Supplemental Material, Table S3. Because the multidose vials of meningococcal and influenza vaccines currently available for pediatric use contain 25 μg EtHg per 0.5 mL dose (Fiore et al. 2008), we purchased multiple single-dose thimerosal-free vaccines and added thimerosal so that the influenza and meningococcal vaccines doses used contained 3.96 μg EtHg per 0.5 mL, as described above. In 2002, the CDC recommended that pregnant women be vaccinated against influenza (Bridges et al. 2002). To replicate this, a single prenatal influenza vaccine containing 25 μg EtHg was administered approximately 4 weeks before estimated delivery to all pregnant dams giving birth to infants assigned to the 2008 study group. All other dams received a single saline injection.

*Vaccine administration*. According to study group assignment, all animals received either a vaccine or saline injection, administered IM, subcutaneously, or by oral gavage, depending on the manufacturer’s recommendations (see Supplemental Material, Table S2). For each IM injection, the needle was inserted at a 90-degree angle and a 0.5 mL dose injected into the left or right biceps femoris of the hamstring. For subcutaneous injections, the skin of the thigh was pinched, the needle inserted at a 45-degree angle, and a 0.5 mL dose administered. When multiple vaccines were to be administered at the same time, different sites within the same area were selected and/or the left and right side alternated.

To adjust the timing of vaccination to human age equivalents, we used a truncated schedule of vaccination. The development of the human and macaque infant visual system is very similar, with the postnatal developmental ratio between the two groups being about 4:1 (Atkinson 1979; Boothe et al. 1980; Teller et al. 1974). This 4:1 ratio is further demonstrated in the development of pattern recognition (Gunderson and Sackett 1984) and the acquisition of object concept permanence (Williams 1979). Thus, the vaccine-dosing schedule was adjusted to accommodate this projected 4:1 developmental trajectory of infant primates.

**Figure 1:**
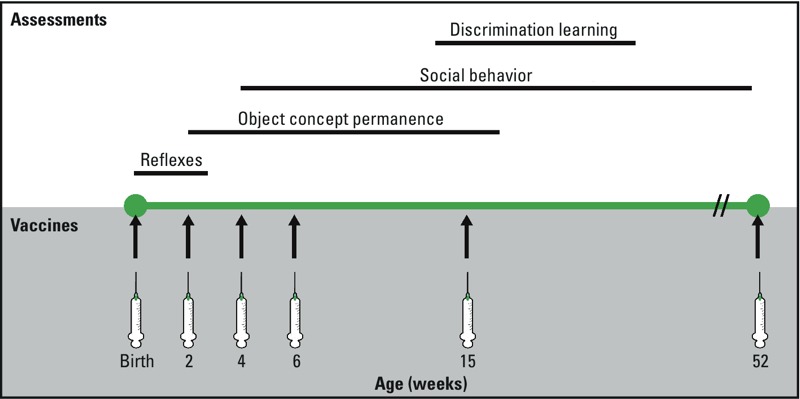
Timing of vaccine administration for the accelerated vaccine schedule in relation to implementation of neurobehavioral assessments.

*Implementation of neurobehavioral assessments*. Assessments of infant development were based on protocols developed at the IPRL and have been extensively published (Burbacher et al. 2013; Chamove and Molinaro 1978; Harlow 1959; Piaget 1954; Sackett et al. 2006a; Schneider and Suomi 1992). All assessments were conducted by three trained testers (see Supplemental Material, Table S4) who were reliability-tested to a minimum 85% agreement every 6–9 months, and who were blinded to the assignment of animals to study groups. Infants underwent developmentally appropriate assessments from birth to 12 months of age. Brief descriptions are given below (for detailed information, see Center on Human Development and Disability 2009). The timing of neurobehavioral assessments in relation to vaccine administration is shown in Figure 1.

*Acquisition of neonatal reflexes*. Infants were assessed for the presence of 19 neonatal reflexes based on the Neonatal Behavioral Assessment Scale (Brazelton 1978). Tests, performed daily from birth to 20 days of age, measured days to criterion for survival reflexes, basic motor reflexes, visual and auditory orienting, muscle tone, and behavioral state (Chamove and Molinaro 1978; Sackett et al. 2006a; Schneider and Suomi 1992).

*Object concept permanence testing*. The object concept permanence (OCP) physical-search test consisted of four tasks: plain reach, screen, well, and A-not-B (Sackett et al. 2006b). The object used as the reward consisted of a small toy covering a grape. The screen and well tasks had three conditions: no hide with the reward in plain view, partial hide with the object half covered, and full hide with the object fully hidden behind the screen or fully covered by a lid over the well. OCP was tested for each infant for 4 days/week from 14 days of age until the infant reached performance criteria on all tasks. Fifteen trials were presented in each session, and data were recorded as the number of sessions to criterion.

*Discrimination/reversal learning and learning set*. Discrimination/reversal testing was initiated at 75 days of age and implemented using a touch screen computer program modeled after the Wisconsin General Testing Apparatus (Harlow and Bromer 1938). Computer testing procedures followed those previously reported (Mandell and Sackett 2008, 2009). Infants were placed in a wire mesh cage with a touch-screen computer monitor mounted vertically to an opening of the cage. An initial adaptation procedure trained the infants to use the touch screen. Training was accomplished through successive approximation by rewarding the infants for approaching, touching, and finally activating the touch screen where a colored stimulus appeared. A stimulus appeared randomly in one of nine possible screen locations. The infant was considered trained when it correctly touched the screen only where the stimulus appeared on 23 of the 25 trials on a single day.

Discrimination and reversal testing immediately followed the adaptation phase and consisted of 25 trials/day. Test trials were a maximum of 60 sec, and the intertrial interval was 10 sec (Mandell and Sackett 2009). Throughout testing, no correction procedure was used. Two stimuli differing only in color were presented in random locations on the screen. A balk was recorded if there was no response within 60 sec after stimulus presentation, which is the accepted method for calculating nonresponsive trials. If the animal balked on 5 trials in a row, the session was terminated.

In the initial discrimination phase, the color of the rewarded stimulus was randomly chosen for each infant. The initial discrimination was run until the infant reached the criterion of 80% correct on a single day. After attaining criterion, the color of the rewarded stimulus image was reversed to the nonrewarded color and 25 trials/day were run again to the same criterion. This was repeated for a total of four reversals. Six animals were removed from the analysis due to experimenter error (1990s Primate, *n* = 4; MMR, *n* = 1; and TCV, *n* = 1). These animals were moved to the first reversal on discrimination learning without reaching criterion. All of these animals were performing above 70% correct when this was done, but they had not yet met the required 80% correct to reach criterion.

Learning set presented the animal with a series of discrimination problems. Each problem had two unique stimulus images, with one randomly selected as the reward image. Each unique problem was presented to the infant for six trials, and then the images were changed to a new problem. Each infant was presented with 6 problems/day and received 240 problems over a minimum of 40 test days. If an infant balked for five trials in a row, that session was terminated. During the study, there was a modification to the software that affected the way the learning set was presented. The spatial distribution of the stimuli changed from three screen locations to nine, potentially increasing the difficulty of this test. Because the majority of animals (*n* = 54) started learning-set testing after this software change, only these animals were included in the analyses (control, *n* = 8; TCV, *n* = 8; MMR, *n* = 12; 1990s Primate, *n* = 8; 1990s Pediatric, *n* = 12; and 2008, *n* = 8). Although the software change did not affect the discrimination/reversal task, the same 54 animals were analyzed for both tasks so that the groups of animals were consistent.

*Social behavior*. Social behavior was evaluated in 40-min daily playroom sessions for each peer group of four animals from approximately 30 days to 12 months of age. The playroom was approximately 2 m wide × 2 m deep × 1.5 m high and contained wire mesh shelves, climbing platforms, and toys. Scoring was conducted by a blinded observer in 5-min focal periods using a coding system of mutually exclusive and exhaustive behaviors (Burbacher et al. 1990; Sackett et al. 1973). The order of testing was randomized for each session. Scored behaviors included passive, explore, withdraw, fear disturbance, rock-huddle-self-clasp, stereotypy, play, sex, and aggression, and could be scored as either a social interaction or a nonsocial behavior (see Supplemental Material, Table S5).

*Statistical analyses*. Neonatal reflexes. The acquisition of neonatal reflexes was coded as the number of days from birth to reaching criterion for a putative reflex. Days to criterion was modeled using Cox regression for reflexes that had a single outcome (snout, suck, righting, or startle) and multilevel Cox regression for all reflexes that were highly correlated (e.g., right- and left-hand grasping). Cox proportional hazards regression models were fitted using the R survival package (Therneau and Grambsch 2000) with Breslow’s method for tied time to events. Mplus software (Muthén and Muthén 2012) was used to fit multilevel Cox regression models with a random intercept for animal, which accounts for the correlation in responses between observations from the same animal. In the event that criterion was not met, the days to criterion was truncated at 21 days and right censored. Condition was dummy coded so that the control group was the reference condition, and vaccine groups were each coded one if an animal participated in a putative condition or zero otherwise. The proportional hazards assumption was assessed for each reflex. The joint null hypothesis that all conditions had identical hazard functions was tested using a likelihood ratio test (LRT) that compared a null model with a model fitted with the experimental conditions, where a significant LRT indicates group differences; the null model for the multilevel Cox model included a random intercept. In the event of a significant LRT, we examined individual parameters to assess whether differences represented differences between the control and a vaccine group. False discovery rate (FDR) corrections to *p*-values were applied across LRTs and within each unique control versus vaccine group (e.g., control vs. TCV) to determine a significance cutoff (Benjamini and Hochberg 1995).

Object permanence. To analyze the development of object permanence, we used a Cox proportional hazards regression, fit in a manner identical to the method described above. In the event that the criterion was not met, days were truncated at 75 and right censored. Condition was dummy coded as described above for the reflex models. LRTs of the joint null hypothesis of identical hazard functions across conditions for object permanence were performed as described above. FDR corrections to *p*-values were applied in the manner as described above.

Discrimination learning. Data were initially summarized as the number of trials to attain 80% criterion on a single test day. We analyzed trials to criterion using survival analysis with Cox regression, and identified median trials to criterion for the control group. This median point was the 25-trial interval, at which the probability of passing was 0.5 for the control group. The probability of passing at this trial interval was calculated for all the other groups, allowing for comparison of the vaccine groups to the midpoint of the survival curve for the control group. Groups with a higher probability of passing than the control group at this trial interval were quicker to attain criterion, whereas groups with a lower probability of passing were slower to attain criterion.

Learning set. Data were cleaned following published procedures (Mandell et al. 2011). Briefly, trials on which the animal balked were removed. If the animal completed fewer than three trials in the problem, the entire problem was excluded from the analysis. All remaining trials and problems were resequenced so that trial 1 in the analysis represents the first attempt at the problem and problem 1 represents the first problem where three or more trials were completed. The resequenced data were then aggregated across 40 problem blocks of the 240 total problems, creating a percentage of correct responses per trial on the problem block. Multilevel modeling was used to analyze the learning-set data, which were fit using an autoregressive covariance structure to reflect the incremental increase in performance that is expected between trials and between problem blocks. Trial, problem block, and group were included as fixed factors, and the intercept was modeled as a random effect. Vaccine groups were compared with the performance control group using the coding procedure described above.

Social behavior. Prior to model building, we examined descriptive statistics for duration and frequency of social and nonsocial behaviors (see Supplemental Material, Table S6). Because duration and frequency were highly correlated, we used only duration as an outcome in the analytic models. Durations of the negative behaviors (withdrawal, fear/disturbance, rock-huddle-self-clasp, and stereotypy) were summed for each animal, as were durations of the positive behaviors (play, sex, and aggression). Thus, for both social (involving one or more animals) and nonsocial (involving no other animal) behaviors, four behavior outcomes were used in the analysis: passive, explore, negative, and positive. A 30-day average was computed for the duration of each of the four nonsocial and social behaviors for each animal for each 30-day period from 30 days to 360 days of age. Duration values were natural log-transformed to reduce the possibility of disproportionate impact of extreme values. Models were fit following longitudinal model-building strategies in which the unconditional growth model (i.e., the average rate of change in a putative outcome) was established by comparing longitudinal models using the Akaike information criterion. No-change, linear, and quadratic models were fit for each outcome. Time was centered at month 2, the first month of the data. The assessment of unconditional growth models indicated that a quadratic model (i.e., change was nonlinear) was the best model for all outcomes, except for a linear trend for social positive behavior. After establishing the growth model for each outcome, we added the intervention condition and an interaction between time parameter and the intervention condition to the models to test for differences in experimental conditions and for differences in developmental trajectory of a putative behavior as a function of experimental condition, respectively. An FDR correction was applied to each parameter across the eight models. In the event of either a significant effect for group or a group × time interaction, we estimated simple slope comparisons (Bauer and Curran 2005) between the control group and each of the vaccine groups. The differences were computed at 2 months and 12 months of age to assess any differences between the experimental groups and the control group at the beginning and at the end of the study period, using an FDR within each time-point.

## Results

*Acquisition of neonatal reflexes*. There were no significant differences between groups in days to criterion for the acquisition of neonatal reflexes except for hand top of counter [Table 3; χ^2^(5 df) = 20.99; *p* = 0.016). This effect was driven by the 1990s Pediatric group [hazard ratio (HR) = 0.36; 95% CI: 0.19, 0.68; *p* = 0.040]. Survival analysis was significant for both left (*z* = –2.80; *p* = 0.005; HR = 0.32; 95% CI: 0.14, 0.71) and right (*z* = –2.07; *p* = 0.038; HR = 0.44; 95% CI: 0.20, 0.96) hand top of counter (see Supplemental Material, Figure S1).

**Table 3:** Likelihood ratio tests for acquisition of neonatal reflexes.

Reflex tested	χ^2^	df	FDR *p*-value
Rooting	3.18	5	0.935
Snout	6.03	5	0.865
Suck	2.27	5	0.935
Startle	2.98	5	0.935
Righting	3.61	5	0.935
Grasp feet	6.94	5	0.749
Clasp	2.06	5	0.935
Functional grasping	5.17	5	0.901
Resistance hands	8.08	5	0.608
Resistance feet	0.94	5	0.967
Hand side of counter	3.79	5	0.935
Feet side of counter	2.23	5	0.935
Hand top of counter	20.99	5	0.016
Feet top of counter	2.97	5	0.935
Auditory orientation	9.09	5	0.608
Visual orientation near	9.09	5	0.608
Visual follow near	1.30	5	0.967
Visual orientation far	5.09	5	0.901
Visual follow far	8.20	5	0.608
df, degrees of freedom.			

*Object concept permanence*. Sessions to criterion for the four stages of object permanence testing are shown in Table 4. No significant differences between groups were observed.

**Table 4:** Likelihood ratio tests of joint null hypothesis of identical hazard functions across conditions for each stage of object concept permanence testing.

Stage of testing	χ^2^	df	FDR *p*-value
Partial reach	2.24	5	0.970
No hide screen	1.06	5	0.970
Partial hide screen	0.91	5	0.970
Full hide screen	9.18	5	0.408
No hide well	3.18	5	0.970
Partial hide well	3.01	5	0.970
Full hide well	12.24	5	0.253
A not B	2.84	5	0.970
df, degrees of freedom.			

*Discrimination/reversal learning*. During the initial two-choice learning phase, there were no significant differences between groups in the number of trials to criterion (Table 5). During the reversal phases, animals in the TCV group achieved criterion in fewer trials than animals in the control group in reversals 2, 3, and 4 [reversal 1: HR = 1.81 (95% CI: 0.99, 3.34), *p* = 0.069; reversal 2: HR = 2.91 (95% CI: 1.45, 5.87), *p* = 0.013; reversal 3: HR = 2.36 (95% CI: 1.24, 4.52), *p* = 0.015; and reversal 4: HR = 2.55 (95% CI: 1.34, 4.88), *p* = 0.013]. The animals in the 1990s Primate group were also significantly more likely to achieve criterion in fewer trials than the control animals except for reversal 3 [reversal 1: HR = 4.39 (95% CI: 2.17, 8.91), *p <* 0.005; reversal 2: HR = 2.46 (95% CI: 1.31, 4.65), *p* = 0.013; reversal 3: HR = 1.07 (95% CI: 0.57, 2.01), *p* = 0.659; and reversal 4: HR = 2.29 (95% CI: 1.19, 4.38), *p* = 0.022]. During reversal testing, the MMR group took longer to achieve criterion during the second reversal (HR = 0.36, *p* = 0.004), but performance was not significantly different from the control group on the other three reversal phases, suggesting that this finding was due to random variation.

**Table 5:** Comparison of performance of control and vaccine groups on discrimination and each reversal phase.

Group	Trial interval^*a*^	Passing probability	Passing SE	HR (95% CI)^*b*^	FDR *p*-value
	Discrimination
Control	125–150	0.54	0.06	—	—
MMR		0.67	0.06	1.72 (1.00, 2.97)	0.103
TCV		0.56	0.07	0.87 (0.47, 1.66)	0.706
1990s Primate		0.49	0.08	1.65 (0.90, 3.05)	0.133
1990s Pediatric		0.40	0.07	0.51 (0.27, 0.96)	0.090
2008		0.56	0.06	0.77 (0.44, 1.35)	0.890
	Reversal 1
Control	200–225	0.56	0.05	—	—
MMR		0.66	0.04	0.91 (0.53, 1.56)	0.764
TCV		0.73	0.04	1.81 (0.99, 3.34)	0.069
1990s Primate		0.83	0.05	4.39 (2.17, 8.91)	0.005
1990s Pediatric		0.64	0.06	0.64 (0.35, 1.24)	0.175
2008		0.56	0.04	0.65 (0.37, 1.10)	0.890
	Reversal 2
Control	200–225	0.56	0.05	—	—
MMR		0.53	0.04	0.36 (0.21, 0.61)	0.004
TCV		0.77	0.05	2.91 (1.45, 5.87)	0.013
1990s Primate		0.73	0.06	2.46 (1.31, 4.65	0.013
1990s Pediatric		0.68	0.05	1.11 (0.64, 1.90)	0.712
2008		0.65	0.04	0.96 (0.51, 1.80)	0.892
	Reversal 3
Control	150–175	0.51	0.06	—	—
MMR		0.52	0.05	1.02 (0.59, 1.74)	0.800
TCV		0.59	0.06	2.36 (1.24, 4.52)	0.015
1990s Primate		0.52	0.06	1.07 (0.57, 2.01)	0.659
1990s Pediatric		0.50	0.06	0.51 (0.28, 0.90)	0.090
2008		0.45	0.04	0.87 (0.46, 1.63)	0.892
	Reversal 4
Control	175–200	0.52	0.05	—	—
MMR		0.47	0.05	0.62 (0.35, 1.10)	0.140
TCV		0.68	0.06	2.55 (1.34, 4.88)	0.013
1990s Primate		0.65	0.06	2.29 (1.19, 4.38)	0.022
1990s Pediatric		0.49	0.06	0.72 (0.42, 1.23)	0.284
2008		0.52	0.04	0.93 (0.47, 1.81)	0.892
HR, hazard ratio. ^***a***^The trial interval is the 25 trial block (test day) where the control group first had > 50% probability of reaching criterion. ^***b***^Hazard ratios test the total number of trials to criterion for each group.					

An error analysis was conducted to assess differences in perseverative behavior between groups. Perseveration was defined as any day of testing that an animal performed < 34% correct or balked on the session, if the balk day was preceded by a perseverative day. All other test days were classified as nonperseveration. Classifying test days in this way has been shown to be sensitive to prefrontal lesions (Jones and Mishkin 1972), as well as to development in humans (Overman et al. 1996) and primates (Mandell and Ward 2011) of a comparable age. A one-way analysis of variance revealed no significant differences between groups for perseverative behavior and balks for any discrimination or reversal phase (see Supplemental Material, Table S7).

*Learning set*. The key outcome in a successful learning-set analysis is a significant 2-way interaction between Block and Trial that shows better performance on trials 2–6 as the animal progresses through testing. Overall, there was not a significant Block × Trial interaction [Table 6; *F*(35, 1606.7) = 0.8, *p* = 0.79], nor was there a significant main effect for Group [*F*(5, 543.1) = 2.03, *p* = 0.07]. Percent correct for the Block × Trial interaction for each group revealed a similar pattern to the overall Block × Trial interaction with no evidence for learning-set formation and only modest within-problem learning by trials 5 and 6 in the later blocks (see Supplemental Material, Figures S2,S3). Although there was a significant three-way interaction (Table 6), the lack of evidence for learning-set formation with any of the groups, as well as the lack of a clear pattern of differences in contrast testing, suggests that this result does not reflect an interpretable learning difference between the groups. Finally, overall latency for the Block × Trial interaction was highest on trial 1 and remained high on subsequent blocks (see Supplemental Material, Figure S4). When we examined the Block × Trial interaction for each study group, we found that all groups had the same general pattern of high reaction times on trial 1(see Supplemental Material, Figure S5). The TCV group had the slowest overall reaction times and was significantly slower than the control group (mean difference, 1.83; 95% CI: 0.96, 2.69). The 2008 group also had reaction times significantly slower than the control group (mean difference, 0.91; 95% CI: 0.12, 1.70).

**Table 6:** Type III test for fixed-effect model results for learning-set performance.

Parameter	*F*-test (df)	*p*-Value
Intercept	37990.9 (1, 534.3)	< 0.001
Problems	5.06 (7, 702.8)	< 0.001
Trials	15.27 (5, 1579.8)	< 0.001
Group	2.03 (5, 543.1)	0.07
Block × Trial	0.80 (35, 1606.7)	0.79
Block × Group	0.57 (35, 701.6)	0.97
Trial × Group	1.18 (25, 1579.8)	0.25
Block × Trial × Group	1.20 (175, 1607.1)	0.04
df, degrees of freedom.		

**Figure 2:**
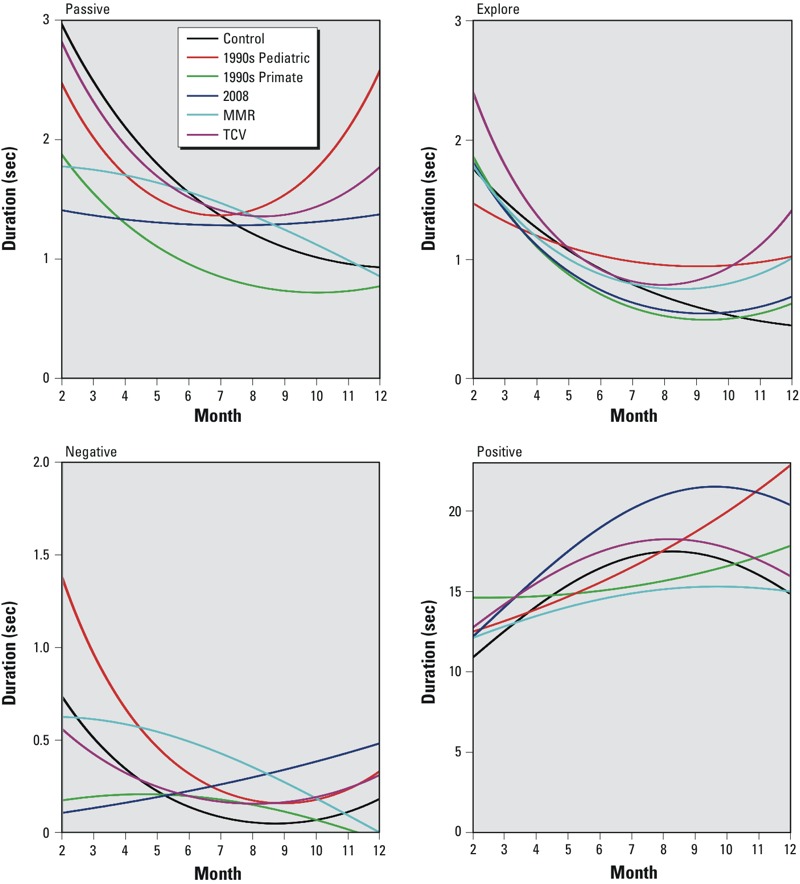
Fitted values from analytical models of social behavior for all groups from 2–12 months of age.

*Social behavior*. Overall means and SDs for duration and frequency of social and nonsocial behaviors scored for all infants is shown in Supplemental Material, Table S6. The duration and frequency of negative behaviors by animals in all groups was very low; in fact, there were no instances of stereotypies recorded across all sessions (see Supplemental Material, Table 6). Analyses of social interaction data identified a significant Group × Quadratic interaction [*F*(5, 752) = 2.92, *p* = 0.030] for negative behaviors, indicating that longitudinal change in negative behaviors differed across groups. Follow-up contrasts indicated that at 2 months of age, relative to the controls, animals in the 1990s Primate and 2008 groups exhibited significantly fewer negative behaviors [*t*(752) = –2.47, *p* = 0.034 and *t*(752) = –2.85, *p* = 0.023], respectively (Figure 2; see also Supplemental Material, Table S8). At 12 months of age, there were no significant differences in behaviors in the vaccine groups compared with the control group.

**Figure 3:**
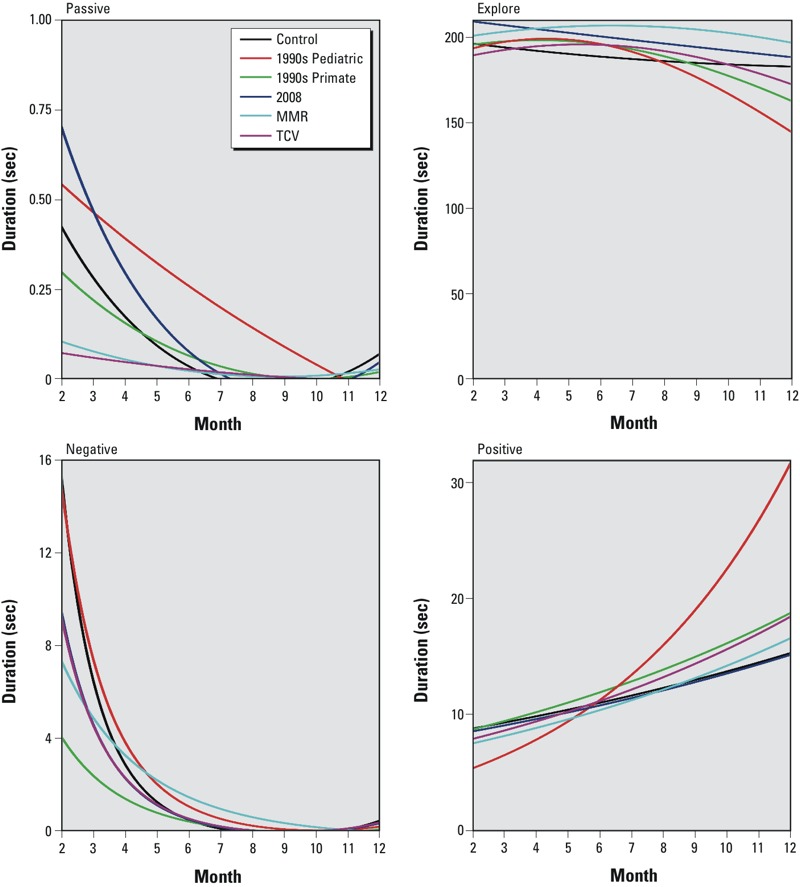
Fitted values from analytical models for non­social behavior for all groups from 2–12 months of age.

Analyses of nonsocial interaction data revealed a significant Group main effect [*F*(5, 211) = 3.62, *p* = 0.011] for passive behaviors. However, animals in the control group exhibited no significant differences in passive behaviors from the experimental groups at both 2 months and 12 months. There was a significant Group × Quadratic interaction [*F*(5, 751) = 3.32, *p* = 0.021] for explore behaviors. Follow-up contrasts indicated that at 12 months of age, relative to the controls, the 1990s Pediatric group exhibited significantly fewer explore behaviors [*t*(751) = –4.62, *p* < 0.001] (Figure 3; see also Supplemental Material, Table S9). There was also a significant Group × Quadratic interaction [*F*(5, 751) = 3.68, *p* = 0.021] for negative behaviors. Follow-up contrasts indicated that at 2 months of age, relative to the control group, the 1990s Primate and MMR groups exhibited significantly fewer negative behaviors [*t*(751) = –4.12, *p* < 0.001] and [*t*(751) = 2.35, *p* = 0.048], respectively. We observed no significant differences in negative behaviors in the vaccine groups relative to the control group at 12 months. There was a significant Group × Linear time interaction [*F*(5, 751) = 13.97, *p* < 0.001] for positive behaviors. Follow-up contrasts indicated that, relative to the control group, the 1990s Pediatric group exhibited significantly fewer positive behaviors [*t*(751) = –2.95, *p* < 0.016] at 2 months, and significantly greater positive behaviors at 12 months [*t*(751) = 4.75, *p* < 0.001] (see Supplemental Material, Table S9).

## Discussion

In this primate study of vaccine safety, we examined a number of neurobehavioral tests—the acquisition of neonatal reflexes, the development of object permanence, the formation of discrimination learning strategies, and assessments of social behavior—in a primate model of vaccine safety. Using a modified version of the Neonatal Behavioral Assessment Scale, we found that days to criterion for the acquisition of neonatal reflexes was similar for animals irrespective of vaccination status, suggesting that auditory and motor function at this age were normal. The only exception was for the acquisition of the hand top of counter reflex for the 1990s Pediatric group, which took longer than the control group. These data are in contrast to our previous pilot study in which a delay in the acquisition of the root, suck, and snout survival reflexes were reported for primate infants following exposure to the birth dose of the thimerosal-containing Hep B vaccine (Hewitson et al. 2010a). This discrepancy is most likely due to the larger number of animals in the present study providing more accurate estimates. Furthermore, in the present study, reflexes were examined from birth to 21 days of age, during which some animals received multiple TCVs (not just a single Hep B vaccine, as was used in the previous study), and yet no detrimental effects on the acquisition of survival reflexes were reported for these animals.

Several rodent studies have examined the effect of thimerosal on auditory and motor function (Berman et al. 2008; Hornig et al. 2004; Olczak et al. 2011; Sulkowski et al. 2012). For example, low-dose thimerosal exposure was found to decrease motor function and increase anxiety in SJL mice, which are susceptible to autoimmunity, but not in C57BL/6J or Balb/c mice (Hornig et al. 2004), suggesting that an altered immune system might confer heightened susceptibility to thimerosal in mice. However, SJL mice are functionally blind as early as 4 weeks of age as a result of retinal degeneration (Chang et al. 2002), and they demonstrate poorer performance in tasks that rely heavily on the visual system (Wong and Brown 2006); therefore, their validity in open field tests, as used in the study by Hornig et al. (2004), is questionable. The timing, dosing, and location of thimerosal injections in rodent studies can also have a significant effect on data outcome. The small size of mouse pups and the limited muscle development at times of IM dosing would have resulted in injections that were a combination of IM and subcutaneous routes (Harry et al. 2004), and any vascular involvement or damage to the hindlimb would have negative implications for tests of motor function. In a study similar to that of Hornig et al. (2004), Berman et al. (2008) examined a number of neurobehavioral outcomes in SJL mice following vaccination with low dose thimerosal. They specifically lowered the vaccine injection volumes and verified at 2–3 days postinjection that there was no vascular damage at the site of injection. In that study, no deficits in tests of social interaction, sensory gating, or anxiety were reported. Although Berman et al. (2008) did report a significant locomotor effect, it was limited to female mice in the open field test only at 4 weeks of age, an age when visual acuity may be diminished (Wong and Brown 2006). Other studies have reported a delay in development of the startle reflex and motor learning (Sulkowski et al. 2012) or a decrease in social behavior (Olczak et al. 2011) in rat pups receiving either subcutaneous or IM injections of thimerosal, respectively. These effects were found only at doses of 200–3,000 μg EtHg/kg/BW, which is 15–500 times the level of EtHg found in pediatric vaccines. Such high doses do not allow for sufficient clearing of EtHg, which has been shown to persist in the rat brain for > 30 days following a single acute IM injection of thimerosal (Olczak et al. 2009). Because much of the rodent data reflects different methodologies and timing and dosing of thimerosal, with adverse effects being found only at very high doses, it is difficult to directly correlate these findings with results of our study.

In the present study, we also examined OCP, discrimination/reversal, learning set, and social behavior. Attainment of object permanence requires some understanding that objects are permanent in space and time and continue to exist when removed from the visual field (Piaget 1954), and has been closely linked to early memory development (Diamond 1990). We found no statistically significant differences between vaccinated and control animals on performance in any phase of the OCP testing. Several primate studies have shown that OCP testing is sensitive to various high-risk conditions, such as prenatal exposure to MeHg, prematurity, low birth weight, and birth asphyxia (Burbacher et al. 1986, 2013).

Two-choice color discrimination tests have been used to evaluate basic learning skills in infant primates for many years (Harlow 1959). Mastery of this task requires the animal to learn a simple discrimination between two identical objects that differ in color. In the present study, we found no significant differences in performance in the discrimination phase across all groups. However, there were two consistent group differences during the reversal phases: Animals in both the TCV and 1990s Primate groups achieved criterion in fewer trials than control animals in three of the four reversal phases, although not the same three reversals. Animals in both groups received similar dosing and timing of TCVs; thus, it appears that animals receiving TCVs on the accelerated schedule demonstrated improved performance during reversal testing. In agreement with this finding, previous studies in macaques have shown that both prenatal and postnatal exposure to MeHg resulted in facilitated learning on this task, as well as a spatial alternation task (Gilbert et al. 1993; Rice 1992). Conversely, animals in the 2008 group, which had a higher cumulative exposure to thimerosal at the time of testing due to both prenatal and postnatal vaccinations, showed no evidence of facilitated learning in any phase of reversal testing.

Several clinical studies have examined the relationship between infant thimerosal exposure from TCVs and pediatric outcome. For example, in a British cohort study examining child development and behavior, Heron et al. (2004) reported that exposure to thimerosal at 3 months of age was inversely associated with hyperactivity and conduct problems, motor development, and requirement for speech therapy. More recently, several studies have reported on the effects of exposure to TCVs and subsequent tests of memory and learning, attention, executive function, language, and motor skills in children at 7–10 years of age (Barile et al. 2012; Mrozek-Budzyn et al. 2012; Thompson et al. 2007; Tozzi et al. 2009). In the original CDC study, Thompson et al. (2007) identified a few significant associations with exposure to thimerosal, but these were small and divided equally between both positive and negative effects. For example, among boys, there was a beneficial association between thimerosal exposure and performance IQ but a detrimental association with both behavioral regulation and motor tics. This analysis was then expanded using measurement models to further assess any associations between thimerosal exposure and neuropsychological outcomes. In the subsequent analysis (Barile et al. 2012), the only consistent finding was an association between early thimerosal exposure and the presence of motor tics in boys. In an Italian cohort, Tozzi et al. (2009) found that greater thimerosal exposure was associated with lower scores in motor function (finger-tapping test) and language (Boston Naming test) only in girls. On the basis of the overall study outcomes, Thompson et al. (2007) and Tozzi et al. (2009) concluded that the pattern of results was consistent with these associations occurring by chance and that exposure had no relation to outcome.

Learning-set formation refers to the learning of visual and other types of discrimination problems progressively more quickly as a function of training on a series of problems (Schrier 1984). In the present study, animals in the TCV group demonstrated increased response latencies in learning-set testing compared with the control group but this was not found in animals in the 1990s Primate group, which received the same EtHg exposure. Furthermore, the TCV group showed little evidence that they had performed at a level, or that their responses had organized into a strategy, that was different from that of controls. In fact, the only performance difference was in the overall mean averaged across all of the blocks and trials, not in their learning across trials or blocks, which is the outcome needed to indicate a learning or strategy difference. In fact, the reported difference was found only in the overall mean averaged across all of the blocks and trials, not in their learning across trials or blocks, which is the outcome needed to indicate a strategy difference.

It is well established that primates who are at high risk for poor developmental outcomes may not develop normal social behaviors characteristic for that species. For example, Burbacher et al. (1990) reported that chronic prenatal exposure to 50 μg/kg/day oral MeHg altered the expression of social behavior in primates, such that exposed infants spent more time being passive and less time engaged in play behaviors with peers. Postnatal exposure to lead (Bushnell and Bowman 1979; Levin et al. 1988) or prenatal exposure to TCDD (2,3,7,8-tetrachlorodibenzo-*p*-dioxin) (Bowman et al. 1989) have also been shown to negatively influence social behavior in macaques. Early differences such as these may translate into enduring social deficits that impact the animal’s ability to interact effectively with other animals into adulthood. In the present study, TCVs did not affect the development of social behaviors characteristic of infant macaques of this age. In all study groups, we observed that each of the four social and nonsocial behaviors developed as expected for normal laboratory-reared macaque infants (Worlein and Sackett 1997). It is particularly relevant that, under the hypothesis that TCVs may impact behavior, there were very few instances of negative behaviors, such as rocking, self-clasping, and stereotypy, reported across the entire infancy period for all groups. This is reassuring because infants would have received the full schedule of TCVs during behavioral testing, representing the period of development at highest risk for neurotoxicity.

Based on the observed toxicokinetics in infant primates receiving low-dose IM thimerosal injections (Burbacher et al. 2005), toxicity following TCV administration would appear unlikely. For example, the half-life of Hg in the blood is 7 days in primates (Burbacher et al. 2005), which is similar to data from comparable studies in mouse pups (Zareba et al. 2007) and human infants (Pichichero et al. 2002, 2008). Furthermore, there is minimal accumulation of Hg in the blood after administration of multiple TCVs (Burbacher et al. 2005; Pichichero et al. 2008), suggesting that Hg is rapidly metabolized and either excreted or deposited in tissue. In primates, the half-life of Hg in the brain following thimerosal exposure is 24 days, more than three times that seen in blood (Burbacher et al. 2005). Accumulation of Hg in the brain of primate infants is therefore likely to occur over time with repeated administration of IM thimerosal (Burbacher et al. 2005), although there is no clear evidence in the literature that this accumulation would directly impact neurobehavioral outcome.

Our study has several limitations. First, studies of low-dose thimerosal exposure in primates have employed an accelerated schedule of exposure similar to that used in rodent studies (Burbacher et al. 2005; Hewitson et al. 2010b). This schedule is based on the theoretical developmental ratio of 4:1, that is, 4 weeks of human development is comparable to 1 week for a primate (Boothe et al. 1985). In the present study we examined neurobehavioral effects of TCVs using both an accelerated vaccine primate schedule and the recommended pediatric schedule, neither of which appeared to affect neurobehavioral outcomes, thus suggesting that the toxicokinetics of EtHg in infant primates is not a limiting factor when using an accelerated schedule of dosing.

Second, we used only male animals in our study, and many clinical studies have reported gender-specific effects of organomercurials (reviewed by Llop et al. 2013). For example, higher exposure to EtHg through vaccination in boys was associated with poorer behavioral regulation and a higher likelihood of motor tics, whereas girls performed significantly better in tests of visual–motor coordination when tested at 7–10 years of age (Thompson et al. 2007). Conversely, prenatal and postnatal exposure to dietary MeHg has been reported to have a negative effect on visuospatial testing at 9 years of age, but only in girls (Davidson et al. 2008).

Finally, because of the large sample size in our study, infants were added to the protocol over several breeding seasons spanning 5 years. There is always a possibility of changes in environmental conditions over time, which is a challenging variable to control for, and therefore a potential limitation to this study. Every care was taken to ensure that all testers remained blinded to study group assignment and that they were reliability trained to the highest standard. Furthermore, neurobehavioral assessments followed very detailed protocols that have been used at this facility for more than three decades (Burbacher and Grant 2012; Burbacher et al. 2013).

## Conclusions

We found no evidence of an adverse impact of vaccination status on early neurodevelopmental measures, including the acquisition of neonatal reflexes and the development of object permanence. This was true for animals receiving TCVs, as well as animals in the 2008 group, which received the expanded pediatric vaccine schedule that is very similar to the currently recommended schedule. Although some animals that received TCVs performed better than controls in the reversal phase of discrimination learning, this association was not consistent across all study groups with thimerosal exposure. Furthermore, response latency on learning-set testing was slowest for animals in the TCV group, but this observation was not mirrored in the 1990s Primate group, which received the same EtHg exposure. Finally, all infants, irrespective of vaccine status, developed the typical social behaviors for their age, with very few instances of negative behaviors reported. Although the data as a whole do not support a consistent adverse effect of TCVs on primate development, factors that may modulate the toxicokinetics and toxicodynamics of thimerosal—such as genetics, sex, birth weight, gestational age, maternal health, and chemical coexposures—should be thoroughly investigated.

## Supplemental Material

(3.6 MB) PDF
