# Erratum: “Examination of the Safety of Pediatric Vaccine Schedules in a Non-Human Primate Model: Assessments of Neurodevelopment, Learning, and Social Behavior”

**DOI:** 10.1289/ehp.124-A11

**Published:** 2016-01-01

**Authors:** Britni Curtis, Noelle Liberato, Megan Rulien, Kelly Morrisroe, Caroline Kenney, Vernon Yutuc, Clayton Ferrier, C. Nathan Marti, Dorothy Mandell, Thomas M. Burbacher, Gene P. Sackett, Laura Hewitson

Environ Health Perspect 123:579–589 (2015); http://dx.doi.org/10.1289/ehp.1408257

Table 1 of this article incorrectly inverted the numbers of animals in the control group and in the 1990s Primate group, listing them as 12 and 16, respectively. The correct number of animals in the control group was 16, and the correct number of animals in the 1990s Primate group was 12. The corrected Table 1 appears in this erratum.

In Table S1, the total number of animals in the control group should have been listed as 16, not 20; the total number of animals in the 2011 N study group should have been listed as 24, not 20. The corrected Table S1 appears in this erratum.

These errors do not affect the analysis, study findings, or interpretation of the results. The authors regret these typographical errors.

**Table 1:** Study groups, sample sizes (*n*), and schedules for vaccine administration.

Group	*n*	Birth	2 weeks	4 weeks	6 weeks	15 weeks	52 weeks
Control	16	Saline	Saline	Saline	Saline	Saline	Saline
			Saline	Saline	Saline	Saline	Saline
			Saline	Saline	Saline	Saline
MMR	15	Saline	Saline	Saline	Saline	MMR	MMR
			Saline	Saline	Saline	Saline	Saline
			Saline	Saline	Saline	Saline
TCV	12	Hep B	Hep B	Hep B	Hep B	Saline	Saline
			DTaP	DTaP	DTaP	DTaP	DTaP
			Hib	Hib	Hib	Hib
1990s Primate	12	Hep B	Hep B	Hep B	Hep B	MMR	MMR
			DTaP	DTaP	DTaP	DTaP	DTaP
			Hib	Hib	Hib	Hib
1990s Pediatric^*a*^	12	Hep B	Hep B	Hep B	Hep B	MMR	None
			DTaP	DTaP	DTaP	DTaP	
			Hib	Hib	Hib	Hib	
2008	12	See Supplemental Material, Table S3, for details					
Abbreviations: Hep B, hepatitis B vaccine; DTaP, diphtheria, tetanus, acellular pertussis vaccine; Hib, Haemophilus ­influenza B vaccine; MMR, measles, mumps, rubella vaccine; TCV, thimerosal-containing vaccines. ^***a***^For the 1990s Pediatric group, vaccines were administered at birth, 2 months, 4 months, 6 months and 15 months; the MMR and DTaP boosters were not administered at 52 months because animals were sacrificed at approximately 18 months.							

**Table S1:** Assignment of animals to each study group across the 5 breeding seasons.

Study Group^*a*^	2008 N	2009 N	2010 N	2011 N	2012 N	Total N
Control	4	4	0	8	0	16
MMR	3	0	4	8	0	15
TCV	0	4	4	4	0	12
1990s Primate	0	4	8	0	0	12
1990s Pediatric	0	0	0	0	12	12
2008	0	4	4	4	0	12
Total	7	16	20	24	12	79
^***a***^Animals in each study group were derived from pregnancies from multiple breeding seasons. For example, animals in the control group were included in years 2008, 2009 and 2011, whereas animals in the TCV group were included in 2009, 2010 and 2011. The only exception to this was made for animals in the 1990s Pediatric group. This group was added to the study protocol in 2011 as a protocol modification, so all pregnancies for this group were derived in the last year of the study (2012).						

