# Supplementary material for: Examination of the Safety of Pediatric Vaccine Schedules in a Non-Human Primate Model: Assessments of Neurodevelopment, Learning, and Social Behavior
Source: Environ Health Perspect. 2015 Feb 18;123(6):579–89. doi: 10.1289/ehp.1408257 (PMC4455585; doi:10.1289/ehp.1408257)
Supplement: (3.6 MB) PDF [file ehp.1408257.s001.508.pdf]

Environ Health Perspect
doi: 10.1289/ehp.1408257
Erratum: http://dx.doi.org/10.1289/ehp.124-A11

Supplemental Material

Examination of the Safety of Pediatric Vaccine Schedules in a Non-Human Primate Model: Assessments of Neurodevelopment, Learning, and Social Behavior

Britni Curtis, Noelle Liberato, Megan Rulien, Kelly Morrisroe, Caroline Kenney, Vernon Yutuc, Clayton Ferrier, C. Nathan Marti, Dorothy Mandell, Thomas M. Burbacher, Gene P. Sackett, and Laura Hewitson

Table S1. Assignment of animals to each study group across the 5 breeding seasons.

Study Group[a]	2008 N	2009 N	2010 N	2011 N	2012 N	Total N
Control	4	4	0	8	0	16
MMR	3	0	4	8	0	15
TCV	0	4	4	4	0	12
1990s Primate	0	4	8	0	0	12
1990s Pediatric	0	0	0	0	12	12
2008	0	4	4	4	0	12
Total	7	16	20	24	12	79

[a]Animals in each study group were derived from pregnancies from multiple breeding seasons. For example, animals in the control group were included in years 2008, 2009 and 2011, whereas animals in the TCV group were included in 2009, 2010 and 2011. The only exception to this was made for animals in the 1990s Pediatric group. This group was added to the study protocol in 2011 as a protocol modification, so all pregnancies for this group were derived in the last year of the study (2012).

Table S2. Vaccine source, ethylmercury (EtHg) content and route of administration.

Vaccine	Trade name (Manufacturer) NDC #	Abbreviation	EtHg content (mg/0.5 ml dose)	Route of Administration
Hepatitis B	Recombivax HB (Merck) 0006-4981-00	Hep B	1.98[a]	IM
Diphtheria, Tetanus, acellular Pertussis	Infanrix (GlaxoSmithKline) 58160-810-46	DTaP	3.97[a]	IM
Haemophilus Influenza B	ActHIB (Sanofi Pasteur) 49281-545-05	Hib	3.96[a]	IM
Measles Mumps Rubella	MMR-II (Merck) 0006-4682-00	MMR	N/A	sub Q
Inactivated Polio vaccine	IPOL (Sanofi Pasteur) 49281-860-10	IPV	N/A	IM
Rotavirus	Rotateq (Merck) 0006-4047-41	Rota	N/A	oral gavage
Pneumococcal 7-valent Conjugate Vaccine	Prevnar (Wyeth) 0005-1970-67	PCV	N/A	IM
Hepatitis A	VAQTA (Merck) 0006-4831-41	Hep A	N/A	IM
Varicellar	Varivax (Merck) 0006-4827-00	Vari	N/A	sub Q
Meningococcal Polysaccharide Vaccine	Menomune (Sanofi Pasteur) 49281-489-05	MCV	3.96	sub Q
Influenza	Fluzone (Sanofi Pasteur) 49281-009-50	Inf	3.96	IM
Influenza	Fluzone (Sanofi Pasteur) 49281-382-15	Inf	25[b]	IM

[a]These vaccines were thimerosal-free for the 2008 schedule. [b]This vaccine was for pregnant dams only.

Abbreviations: IM, intramuscular; sub Q, subcutaneous.

Table S3. The 2008 pediatric vaccination schedule with the timing adjusted for infant primates[a,].

Timing of Vaccine Administration	Vaccinations Administered
Prenatal	Influenza[b]
Birth	Hep B
2 weeks	Hep B, DTaP, Rota, Hib, PCV, IPV
4 weeks	DTaP, Rota, Hib, PCV, IPV
6 weeks	Hep B, DTaP, Rota, Hib, PCV, IPV, Influenza
12 weeks	Hib, PCV, Hep A
15 weeks	DTaP, MMR, Varicella
18 weeks	Hep A, Influenza
26 weeks	MCV[c]
30 weeks	Influenza
42 weeks	Influenza
52 weeks	DTaP, IPV, MMR, Varicella
54 weeks	Influenza
66 weeks	Influenza
78 weeks	Influenza

[a]The timing of all vaccine administration was accelerated approximately 4:1 to account for the faster developmental trajectory of infant primates. [b]A single pre-natal influenza vaccine containing 25 μg EtHg was administered to all pregnant dams giving birth to infants assigned to this study group approximately 4 weeks prior to estimated delivery. All other dams received a single saline injection. An influenza vaccine containing 3.96 μg EtHg was administered to all infants at 6 weeks of age and then every 12 weeks thereafter, mimicking the pediatric schedule of annual influenza vaccination. [c]The meningococcal vaccine is recommended for certain high-risk groups and was included to maximize potential thimerosal exposure in animals in this study group. This meningococcal vaccine was formulated to contain 3.96 μg EtHg. Abbreviations: Hep B, Hepatitis B vaccine; DTaP, Diphtheria, Tetanus, acellular Pertussis vaccine; Rota, rotavirus vaccine; Hib, Haemophilus influenza B vaccine; MMR, Measles Mumps Rubella vaccine; PCV, pneumococcus vaccine; IPV, inactivated polio vaccine; Varicella, chicken pox vaccine; Hep A, hepatitis A vaccine; MCV, meningococcal vaccine.

Table S4. The distribution of animals and number of test sessions among the three testers for all of the nursery assessments during the 5 years of this study.

Study Group	Tester 1 Animals	Tester 1 Sessions	Tester 2 Animals	Tester 2 Sessions	Tester 3 Animals	Tester 3 Sessions
Saline	15	152	7	53	12	65
MMR	14	119	8	71	10	51
TCV	9	90	0	0	8	39
1990 Primate	12	134	0	0	12	59
1990 Pediatric	12	124	12	77	4	4
2008	12	126	4	46	8	44
Total	74	745	31	247	54	262

Table S5. Description of social and non-social behavioral categories scored for all infants during playroom sessions.

**Behavior**[a]	**Description of Behaviors**
Passive	No intense interaction with other animals, self, or objects. Can include a slow visual scanning component, social contact such as huddling, or proximity within one foot, and occurs without locomotion.
Explore	Visual and/or tactual inspection of other animals, self, or objects, with or without locomotion.
Withdraw	Retreat from an animal or object creating increased distance by locomotion, but with no fear behaviors.
Fear-Disturbance	Fear display involving submissive posture, retraction of lips, cooing, screeching, convulsive jerking, or three successive hoots, with or without withdrawal or locomotion.
Rock-Huddle-Self-clasping	Strong clasping/grasping of another monkey without play behavior, or self-clasping with arms, legs, hands, or feet, without locomotion and no active inspection of own or other's body.
Stereotypy	Repetitive body movements, with or without locomotion, requiring three or more consecutive, repetitive movements.
Play	Behaviors with greater physical intensity than explore, involving 'ears back-mouth puckering' expression, open mouth without teeth exposure or ears back, chasing, wrestling, bouncing, running or jumping, rolling, biting without injury, or 'tug-of-war' with an object.
Sex	Presenting rear area, inspection of genitalia, masturbation, with thrusting toward another animal or tester, mounting and thrusting an animal or object.
Aggression	'Stiff' stance, piloerection, open-mouth threat, back, rolling and hitting with or without injury.

[a]Behaviors can be either interactive with other animals (social behavior) or individual behaviors not involving any other animal (non-social behavior).

Table S6. Overall means and standard deviations for duration and frequency of social and non-social behaviors scored for all infants during playroom sessions.

Behavior	Social Duration (SD)[a]	Social Frequency (SD)	Non-social Duration (SD)[a]	Non-social Frequency (SD)
Passive	2.48 (1.94)	0.58 (0.37)	0.49 (1.60)	0.02 (0.06)
Explore	1.26 (0.73)	0.44 (0.21)	192.99 (16.87)	30.61 (3.59)
Withdrawal	0.03 (0.09)	0.01 (0.03)	0.00 (0.00)	0.00 (0.00)
Fear/Disturbance	0.40 (0.55)	0.10 (0.14)	5.50 (6.23)	0.96 (0.91)
Rock/Huddle/Self-clasp	0.57 (3.12)	0.02 (0.10)	0.12 (0.66)	0.01 (0.04)
Stereotypy	0.00 (0.00)	0.00 (0.00)	0.00 (0.00)	0.00 (0.00)
Play	17.23 (5.87)	5.25 (1.50)	13.86 (3.89)	10.67 (2.19)
Sex	1.54 (1.81)	0.28 (0.31)	0.00 (0.00)	0.00 (0.00)
Aggression	0.03 (0.08)	0.01 (0.02)	0.00 (0.00)	0.00 (0.00)

[a]Duration reported in seconds.

Table S7. Mean number of test days during 2-choice discrimination spent in the perseveration-balk phase[a].

Group	Disc	Rev 1	Rev 2	Rev 3	Rev 4	Total
Control						
Mean	2.75	5.38	5.13	2.75	2.13	3.63
SD	2.43	3.07	2.36	1.043	1.13	2.46
N	8	8	8	8	8	40
TCVs						
Mean	1.125	4.75	6.375	2.75	1.625	3.33
SD	2.03	3.20	5.26	1.67	1.51	3.53
N	8	8	8	8	8	40
MMR						
Mean	1	4.92	5.83	3.42	2.42	3.52
SD	1.28	3.34	5.01	1.88	3.34	3.58
N	12	12	12	12	12	60
1990s Primate						
Mean	0 .88	3.25	2.50	2.5 0	2.25	2.28
SD	1.36	1.91	1.51	3.12	1.67	2.06
N	8	8	8	8	8	40
1990s Pediatric						
Mean	0.83	3.167	4.50	2.00	2.83	2.67
SD	1.40	2.17	2.07	1.35	1.11	2.03
N	12	12	12	12	12	60
2008						
Mean	0.50	7.63	4.38	2.63	1.86	2.50
SD	1.07	9.55	1.92	1.06	1.68	2.83
N	8	8	8	8	7	39
One-Way ANOVAs						
F	1.98	1.21	1.31	0.77	0.43	
p	0.10	0.32	0.27	0.58	0.82	
R^2	0.08	0.02	0.03	0.01	0.01	

[a]Perseveration was defined as any day that an animal continued to choose the incorrect response on 66% or more trials, while balk days were recorded when more than 5 trials exceeded the 60-second response time limit. Perseveration is one of the major error factors in two-choice performance and refusal to perform is often indicative of either boredom or frustration at failure of the choice response to produce reward. Abbreviations: Disc, discrimination; Rev, reversal.

Table S8. Comparison of simple slope differences in social behavior between the control and vaccine groups at 2 and 12 months of age.

Group (vs. Controls)	Simple slope Difference (SE) Month 2	*t* Month 2	*p*-FDR Month 2	Simple slope Difference (SE) Month 12	*t* Month 12	*p*-FDR Month 12
**Social Passive**						
1990s Primate	-0.32 (0.25)	-1.27	0.339	-0.08 (0.25)	-0.33	0.879
1990s Pediatric	-0.13 (0.25)	-0.52	0.750	0.62 (0.25)	2.43	0.076
2008	-0.50 (0.25)	-1.98	0.239	0.21 (0.25)	0.81	0.695
MMR	-0.36 (0.25)	-1.42	0.339	-0.04 (0.27)	-0.15	0.879
TCV	-0.04 (0.25)	-0.15	0.881	0.36 (0.26)	1.41	0.394
**Social Explore**						
1990s Primate	0.04 (0.15)	0.24	0.948	0.12 (0.15)	0.80	0.425
1990s Pediatric	-0.11 (0.15)	-0.75	0.948	0.34 (0.15)	2.23	0.065
2008	0.02 (0.15)	0.12	0.948	0.15 (0.15)	1.03	0.379
MMR	0.01 (0.15)	0.06	0.948	0.33 (0.16)	2.05	0.068
TCV	0.21 (0.15)	1.40	0.814	0.51 (0.15)	3.37	0.004
**Social Negative**						
1990s Primate	-0.39 (0.16)	-2.47	0.034	-0.21 (0.16)	-1.30	0.485
1990s Pediatric	0.32 (0.16)	2.00	0.076	0.12 (0.16)	0.74	0.523
2008	-0.45 (0.16)	-2.85	0.023	0.23 (0.16)	1.41	0.485
MMR	-0.07 (0.16)	-0.41	0.679	-0.17 (0.17)	-0.96	0.523
TCV	-0.11 (0.16)	-0.67	0.626	0.10 (0.16)	0.64	0.523
**Social Positive**						
1990s Primate	0.27 (0.19)	1.47	0.596	0.17 (0.19)	0.93	0.587
1990s Pediatric	0.13 (0.19)	0.69	0.596	0.41 (0.19)	2.19	0.145
2008	0.10 (0.19)	0.55	0.596	0.30 (0.19)	1.59	0.278
MMR	0.10 (0.19)	0.53	0.596	0.01 (0.20)	0.05	0.960
TCV	0.15 (0.19)	0.78	0.596	0.07 (0.19)	0.35	0.903

Table S9. Comparison of simple slope differences in non-social behavior between the control and vaccine groups at 2 and 12 months of age.

Group (vs. Controls)	Simple slope Difference (SE) Month 2	*t* Month 2	*p*-FDR Month 2	Simple slope Difference (SE) Month 12	*t* Month 12	*p*-FDR Month 12
**Social Passive**						
1990s Primate	-0.09 (0.13)	-0.73	0.539	-0.05 (0.13)	-0.37	0.868
1990s Pediatric	0.08 (0.13)	0.61	0.539	-0.12 (0.13)	-0.92	0.868
2008	0.18 (0.13)	1.39	0.274	-0.02 (0.13)	-0.17	0.868
MMR	-0.26 (0.13)	-1.98	0.121	-004 (0.14)	-0.29	0.868
TCV	-0.28 (0.13)	-2.22	0.121	-0.08 (0.13)	-0.57	0.868
**Social Explore**						
1990s Primate	-0.00 (0.05)	-0.06	0.952	-0.12 (0.05)	-2.29	0.055
1990s Pediatric	-0.01 (0.05)	-0.29	0.952	-0.23 (0.05)	-4.62	<0.001
2008	0.06 (0.05)	1.27	0.952	0.03 (0.05)	0.58	0.565
MMR	0.02 (0.05)	0.45	0.952	0.07 (0.05)	1.35	0.297
TCV	-0.04 (0.05)	-0.70	0.952	-0.06 (0.05)	-1.14	0.316
**Social Negative**						
1990s Primate	-1.17 (0.28)	-4.12	<0.001	-0.32 (0.29)	-1.10	0.680
1990s Pediatric	-0.03 (0.28)	-0.09	0.926	-0.20 (0.29)	-0.70	0.805
2008	-0.44 (0.28)	-1.56	0.148	-0.08 (0.29)	-0.29	0.841
MMR	-0.67 (0.29)	-2.35	0.048	-0.42 (0.31)	-1.35	0.680
TCV	-0.47 (0.28)	-1.64	0.148	-0.06 (0.29)	-0.20	0.841
**Social Positive**						
1990s Primate	-0.01 (0.15)	-0.05	0.957	0.19 (0.15)	1.31	0.383
1990s Pediatric	-0.43 (0.15)	-2.95	0.016	0.70 (0.15)	4.75	<0.001
2008	-0.03 (0.15)	-0.17	0.957	-0.01 (0.15)	-0.06	0.950
MMR	-0.14 (0.15)	-0.96	0.846	0.08 (0.15)	0.51	0.765
TCV	-0.10 (0.15)	-0.66	0.850	0.18 (0.15)	1.20	0.383

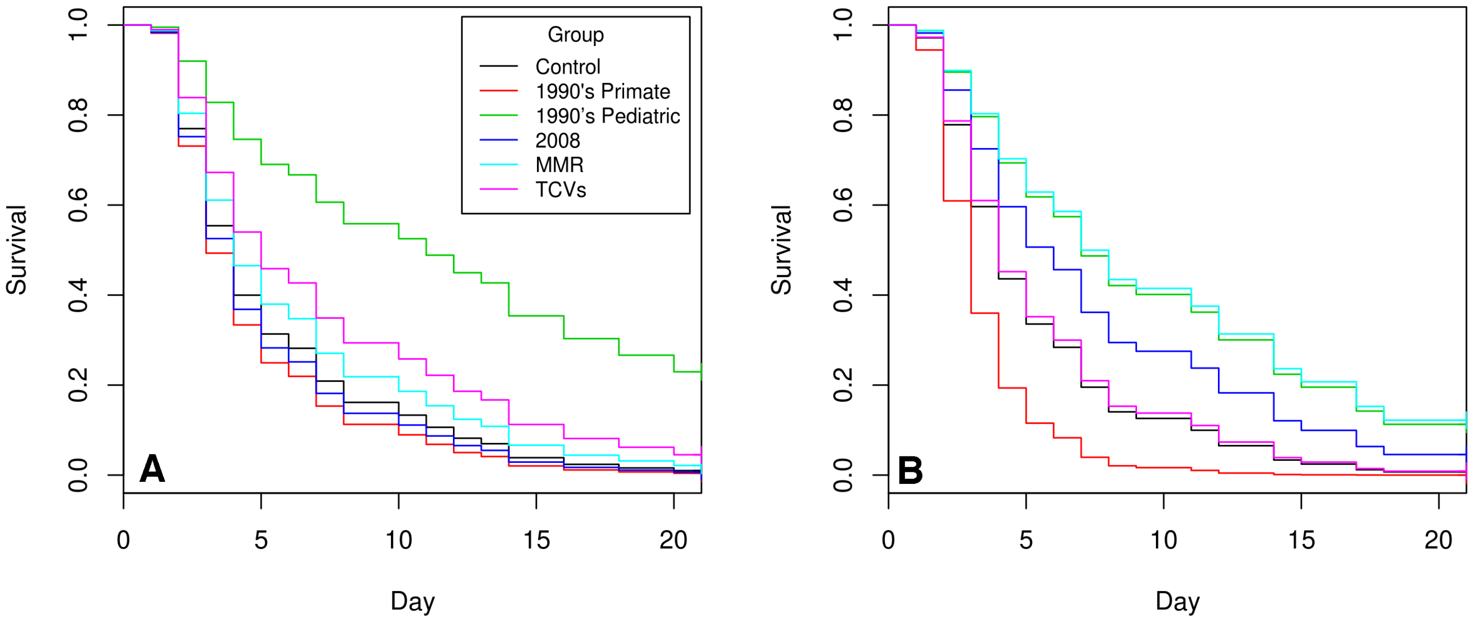

Figure S1. Kaplan–Meier survival curves demonstrating the estimated proportion of animals having reached criterion for the left (A) and right (B) *Hand Top of Counter* reflex. The acquisition of this reflex was scored as time-to-criterion measured daily from birth to 21 days. Animals in the 1990s Pediatric Group took longer to achieve this reflex compared to controls for both the left (z=-2.80, p=0.005, HR=0.32) and right (z=-2.07, p=0.038, HR=0.44) side. Animals in the MMR group also took longer to achieve this reflex but only for the right side (z=-2.11, p=0.035, HR=0.42).

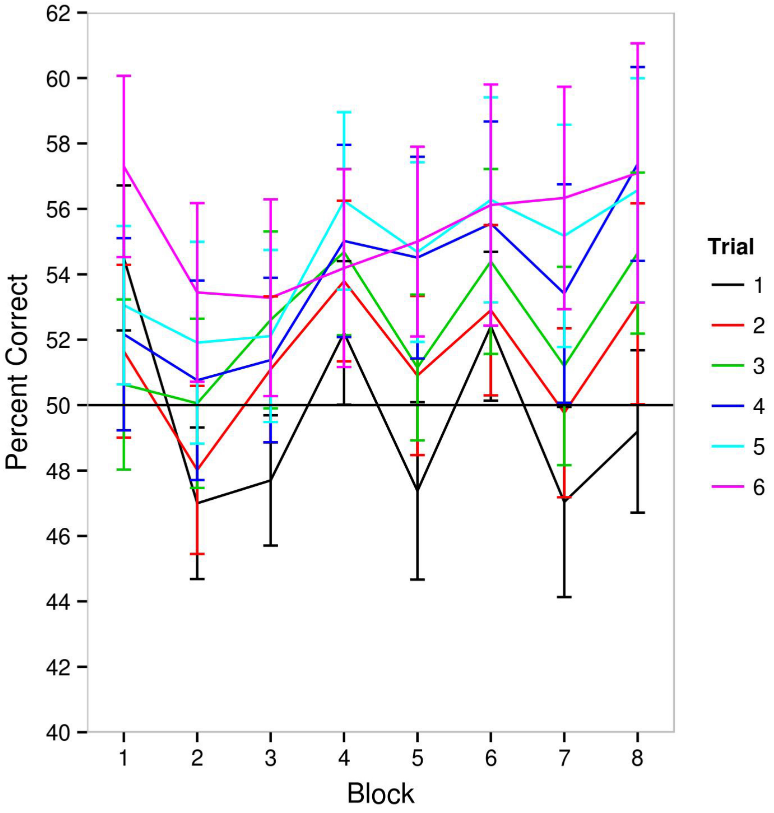

Figure S2. Learning Set data showing percent correct for the overall Block X Trial interaction. For the initial trial of each problem, because either stimulus image may be correct, the performance of the infant is simply a guess. However, as tests advance, the probability of being correct on trial 2 can increase if the infant begins to adopt the concept of 'win-stay' or 'lose-shift' based on the outcome of trial 1. Thus, if this between-problem concept develops, it is expected that the percent of correct responses should increase over blocks of trials beginning with trial 2. The more traditional within-problem 'associative' learning is assessed by the percent of correct responses on trials 2-6. Performance on trial 2 shows little, if any, evidence for formation of a learning set over the 240 problems, while performance on trial 6 indicates only modest within-problem associative learning by trial 6 of the problem on all 8 blocks. The reason for poor performance is uncertain, although this is a difficult task for infant macaques at this age (Mandell and Sackett 2009). Bars, 95% CI.

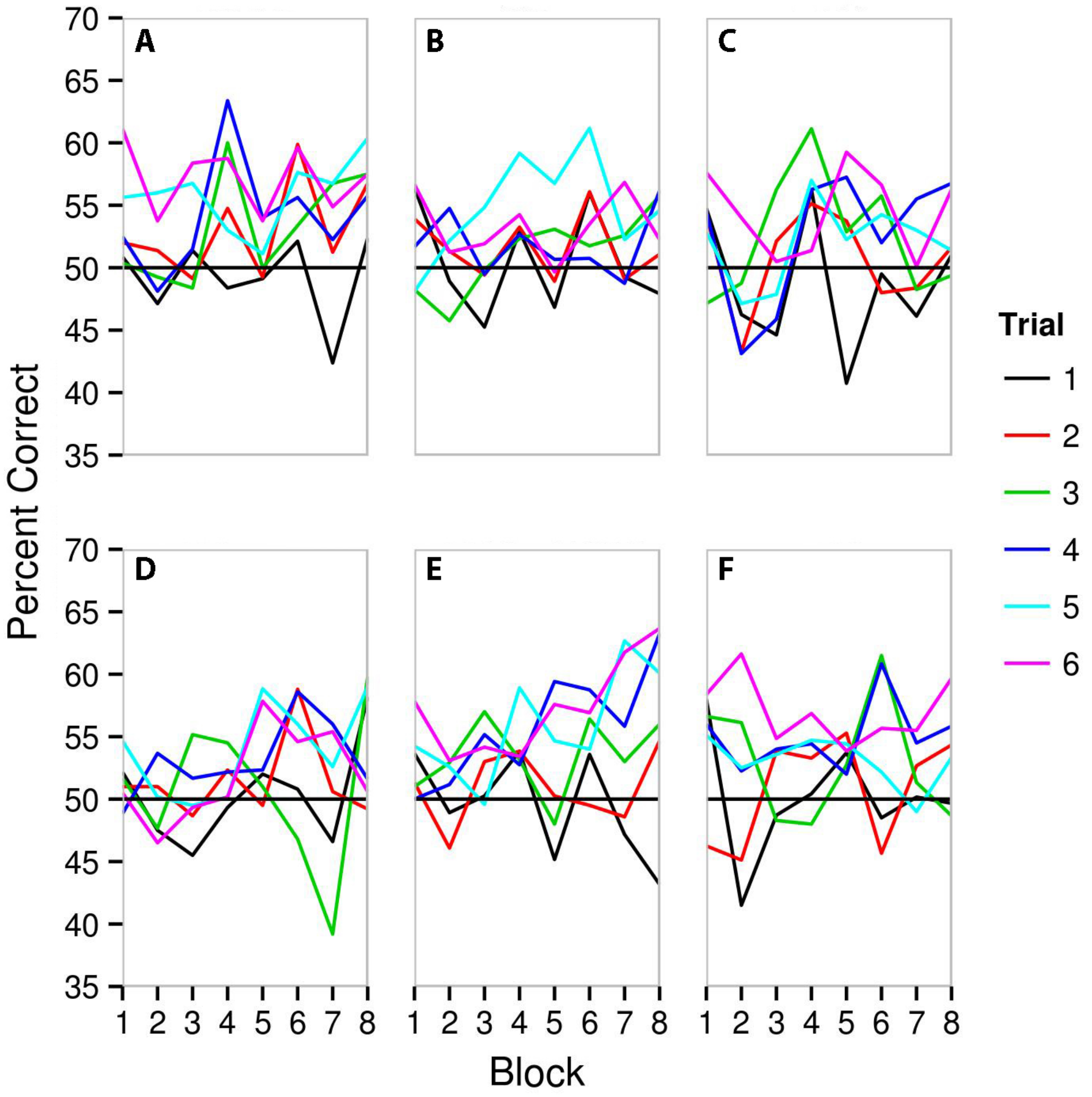

Figure S3. Learning set data showing percent correct for the Block X Trial interaction by each study group. All groups exhibited the general pattern of the overall Block X Trial interaction; namely, there was no evidence for learning set formation, with modest within-problem learning shown by Trials 5 and 6 in the later blocks. A, Control; B, MMR; C, TCV; D, 1990's Primate; E, 1990's Pediatric and F, 2008.

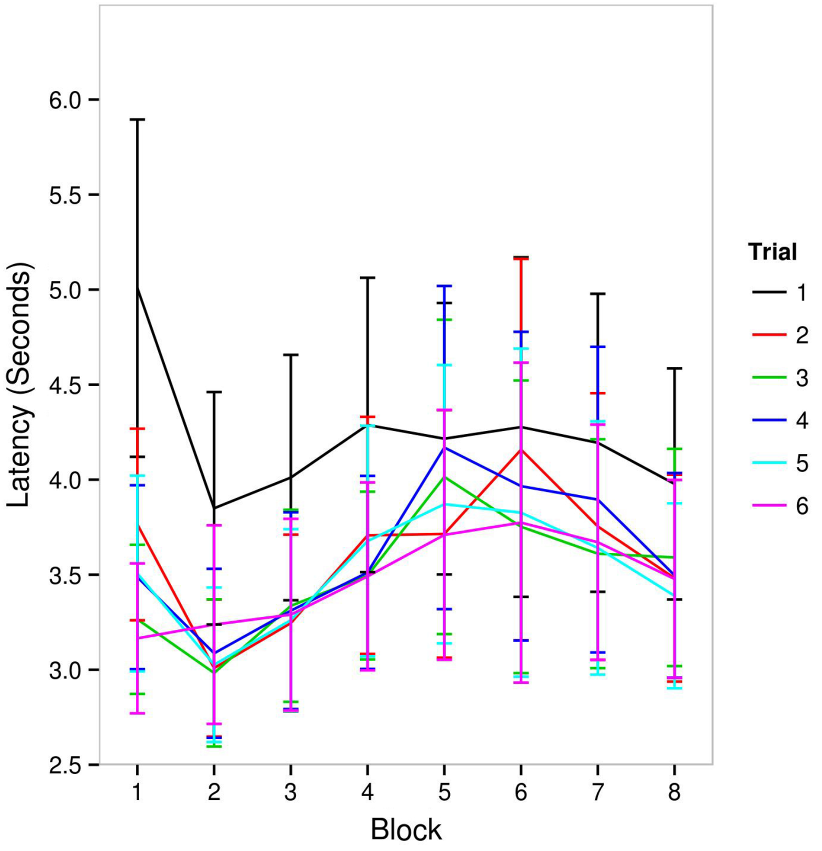

Figure S4. Learning Set data showing overall mean latency (choice reaction time) in seconds for the Block X Trial interaction. As expected, latency was highest on trial one of block one and remained high on subsequent blocks, indicating increased attention to the novel stimuli appearing on the first trials, relative to later trials, of all problems. Bars, 95% CI.

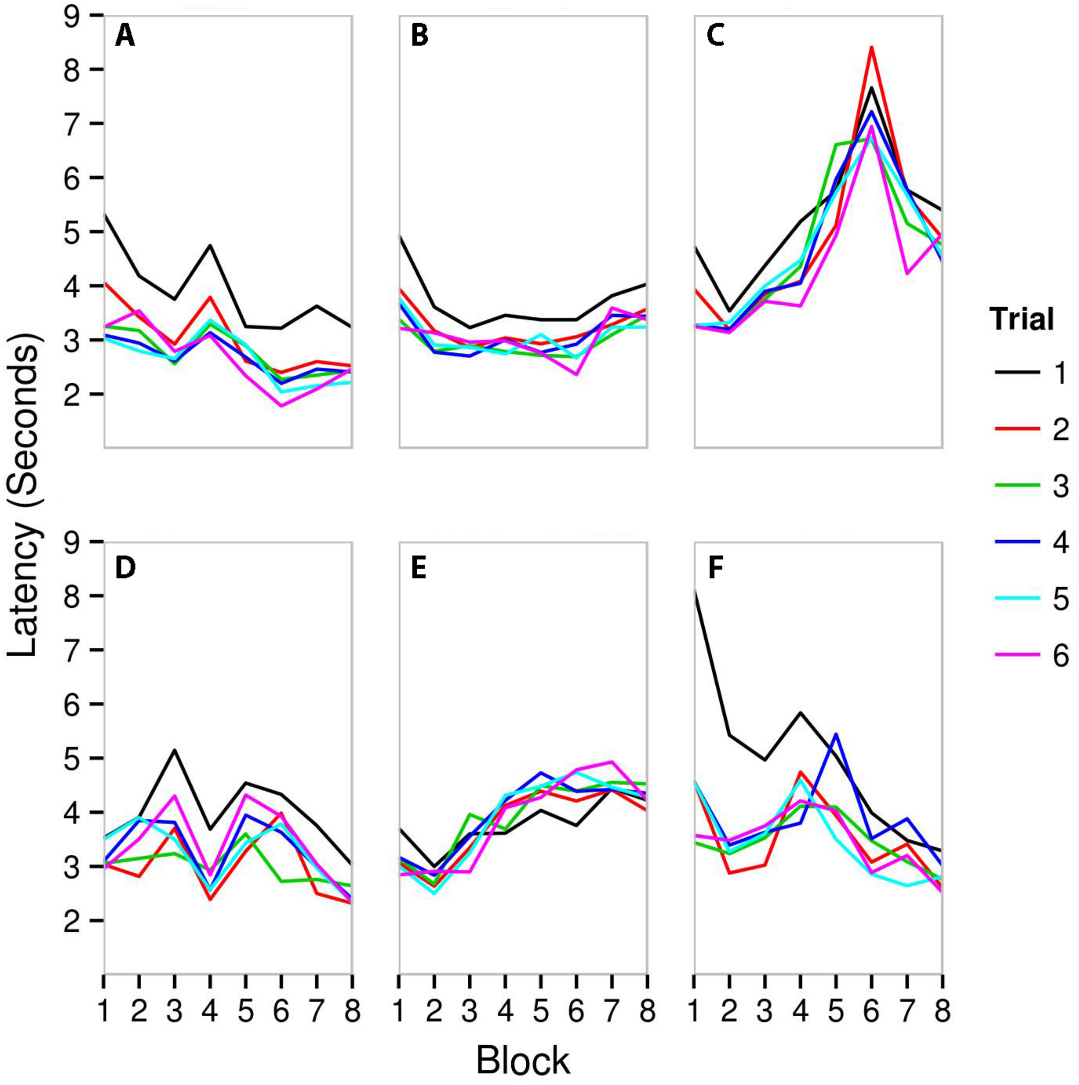

Figure S5. Learning Set data showing mean latency (choice reaction times) in seconds for the Block X Trial interaction by each study group. All groups except TCV and 1990s Pediatric had the general pattern of high trial 1 reaction times with leveling or decreasing reaction times over trials. For these two groups, reaction times increased over trials and blocks. This may indicate a lack of motivation by animals in the TCV and 1990s Pediatric groups, although that was not reflected in their percent correct responses in any obvious way. A, Control; B, MMR; C, TCV; D, 1990's Primate; E, 1990's Pediatric and F, 2008.

Erratum: "Examination of the Safety of Pediatric Vaccine Schedules in a Non-Human Primate Model: Assessments of Neurodevelopment, Learning, and Social Behavior"

Britni Curtis, Noelle Liberato, Megan Rulien, Kelly Morrisroe, Caroline Kenney, Vernon Yutuc, Clayton Ferrier, C. Nathan Marti, Dorothy Mandell, Thomas M. Burbacher, Gene P. Sackett, and Laura Hewitson

Environ Health Perspect 123:579–589 (2015); http://dx.doi.org/10.1289/ehp.1408257

Table 1 of this article incorrectly inverted the numbers of animals in the control group and in the 1990s Primate group, listing them as 12 and 16, respectively. The correct number of animals in the control group was 16, and the correct number of animals in the 1990s Primate group was 12. The corrected Table 1 appears in this erratum.

In Table S1, the total number of animals in the control group should have been listed as 16, not 20; the total number of animals in the 2011 N study group should have been listed as 24, not 20. The corrected Table S1 appears in this erratum.

These errors do not affect the analysis, study findings, or interpretation of the results. The authors regret these typographical errors.

Table 1. Study groups, sample sizes (*n*), and schedules for vaccine administration.

Group	*n*	Birth	2 weeks	4 weeks	6 weeks	15 weeks	52 weeks
Control	16	Saline	Saline<br>Saline<br>Saline	Saline<br>Saline<br>Saline	Saline<br>Saline<br>Saline	Saline<br>Saline<br>Saline	Saline<br>Saline
MMR	15	Saline	Saline<br>Saline<br>Saline	Saline<br>Saline<br>Saline	Saline<br>Saline<br>Saline	MMR<br>Saline<br>Saline	MMR<br>Saline
TCV	12	Hep B	Hep B<br>DTaP<br>Hib	Hep B<br>DTaP<br>Hib	Hep B<br>DTaP<br>Hib	Saline<br>DTaP<br>Hib	Saline<br>DTaP
1990s Primate	12	Hep B	Hep B<br>DTaP<br>Hib	Hep B<br>DTaP<br>Hib	Hep B<br>DTaP<br>Hib	MMR<br>DTaP<br>Hib	MMR<br>DTaP
1990s Pediatric[a]	12	Hep B	Hep B<br>DTaP<br>Hib	Hep B<br>DTaP<br>Hib	Hep B<br>DTaP<br>Hib	MMR<br>DTaP<br>Hib	None
2008	12	See Supplemental Material, Table S3, for details					

Abbreviations: Hep B, hepatitis B vaccine; DTaP, diphtheria, tetanus, acellular pertussis vaccine; Hib, Haemophilus influenza B vaccine; MMR, measles, mumps, rubella vaccine; TCV, thimerosal-containing vaccines.
[a]For the 1990s Pediatric group, vaccines were administered at birth, 2 months, 4 months, 6 months and 15 months; the MMR and DTaP boosters were not administered at 52 months because animals were sacrificed at approximately 18 months.

Table S1. Assignment of animals to each study group across the 5 breeding seasons.

Study Group[a]	2008 N	2009 N	2010 N	2011 N	2012 N	Total N
Control	4	4	0	8	0	16
MMR	3	0	4	8	0	15
TCV	0	4	4	4	0	12
1990s Primate	0	4	8	0	0	12
1990s Pediatric	0	0	0	0	12	12
2008	0	4	4	4	0	12
Total	7	16	20	24	12	79

[a]Animals in each study group were derived from pregnancies from multiple breeding seasons. For example, animals in the control group were included in years 2008, 2009 and 2011, whereas animals in the TCV group were included in 2009, 2010 and 2011. The only exception to this was made for animals in the 1990s Pediatric group. This group was added to the study protocol in 2011 as a protocol modification, so all pregnancies for this group were derived in the last year of the study (2012).
